# The Protective Effect of a Long-Acting and Multi-Target HM-3-Fc Fusion Protein in Rheumatoid Arthritis

**DOI:** 10.3390/ijms19092683

**Published:** 2018-09-10

**Authors:** Ruijing Huang, Jian Li, Yibo Wang, Lihua Zhang, Xiaohui Ma, Hongyu Wang, Wenlei Li, Xiaodan Cao, Hanmei Xu, Jialiang Hu

**Affiliations:** 1 State Key Laboratory of Natural Medicines, Ministry of Education, China Pharmaceutical University, Nanjing 210009, China; huangruijing1981@sina.com; 2 The Engineering Research Center of Peptide Drug Discovery and Development, China Pharmaceutical University, Nanjing 211198, China; 3 Centre for Biopharmaceutical Products, Tasly Pharmaceuticals Co., Ltd., Tianjin 300410, China; lijian16@tasly.com (J.L.); wangyibo@tasly.com (Y.W.); maxiaohui@tasly.com (X.M.); wanghongyu@tasly.com (H.W.); liwenlei@tasly.com (W.L.); caoxd@tasly.com (X.C.); 4 Tasly Academy, Tasly Holding Group Co., Ltd., Tianjin 300410, China; zhanglihua@tasly.com

**Keywords:** rheumatoid arthritis, HM-3, Fc-domain of immunoglobulin G4, synovial angiogenesis, inflammatory response, TNF-α, half-life, pharmacodynamics

## Abstract

Current treatment of rheumatoid arthritis (RA) is limited by relative shortage of treatment targets. HM-3 is a novel anti-RA polypeptide consisting of 18 amino acids with integrin αVβ3 and α5β1 as targets. Previous studies confirmed that HM-3 effectively inhibited the synovial angiogenesis and the inflammatory response. However, due to its short half-life, the anti-RA activity was achieved by frequent administration. To extend the half-life of HM-3, we designed a fusion protein with name HM-3-Fc, by combination of modified Fc segment of immunoglobulin 4 (IgG4) with HM-3 polypeptide. In vitro cell experiments demonstrated that HM-3-Fc inhibited the proliferation of splenic lymphocytes and reduced the release of TNF-α from macrophages. The pharmacodynamics studies on mice paw in Collagen-Induced Arthritis (CIA) model demonstrated that HM-3-Fc administered once in 5 days in the 50 and 25 mg/kg groups, or once in 7 days in the 25 mg/kg group showed a better protective effect within two weeks than the positive control adalimumab and HM-3 group. Preliminary pharmacokinetic studies in cynomolgus confirmed that the in vivo half-life of HM-3-Fc was 15.24 h in comparison with 1.32 min that of HM-3, which demonstrated that an Fc fusion can effectively increase the half-life of HM-3 and make it possible for further reduction of subcutaneous injection frequency. Fc-HM-3 is a long-acting active molecule for RA treatment.

## 1. Introduction

Rheumatoid arthritis (RA), a multi-systemic autoimmune disease, is characterized by joint synovitis, pannus formation and symmetrical destructive joint disease [1]. The microenvironment of RA involves hypoxia within the joint cavity with a large number of inflammatory factors and angiogenic active molecules [2]. These factors together contribute to the formation of characteristic angiogenesis [3,4]. It provides nutrients for proliferating synovial cells and it infiltrates the lubricating membrane with more inflammatory cells and mediators as well, maintaining and promoting the abnormal angiogenesis. Therefore, RA is considered as a vicious cycle with “inflammation-angiogenesis” process and it is called an “angiogenesis disease” [5,6].

HM-3 is an anti-angiogenic polypeptide with 18 amino acid residues, which is generated by the connection of an integrin-targeting RGD (Arg-Gly-Asp) sequence to the C-terminus of an endostatin fragment (IVRRADRAAVP). It targets integrin αvβ3 and α5β1 [7]. Several studies have shown that as an integrin inhibitor, HM-3 directly reduced the expression of vascular endothelial growth factor (VEGF) and platelet derived growth factor A (PDGF-A) in endothelial cells and down-regulated the corresponding signal transduction pathways [8]. HM-3 achieved its anti-RA activity via the anti-inflammatory and anti-angiogenic effects. And it exhibited anti-RA effects in both adjuvant-induced and collagen-induced arthritis models [9]. PEGylated HM-3 (PEG-HM-3) also possessed anti-angiogenesis and anti-rheumatic activity [10]. In vitro, it decreased splenocyte viability and the levels of tumor necrosis factor-α (TNF-α) in macrophage supernatant. It also decreased the expression of toll-like receptor (TLR-4) protein in LPS-induced synoviocytes. In the adjuvant-induced arthritis model, mPEG-SC20_K_-HM-3 (PEG-HM-3) treatment decreased the levels of IL-6 in spleens, TNF-α, cluster of differentiation 31 (CD31) and CD105 in the joint cavity by immunohistochemistry analysis [10]. Therefore, HM-3 and PEG-HM-3 are novel and promising multi-target anti-RA molecules.

However, as a polypeptide, HM-3 is naturally prone to enzymatic hydrolysis by proteolytic enzymes in vivo and it has a short half-life [11]. In vivo pharmacokinetic studies showed that the half-life of HM-3 is only 27 min in male Sprague-Dawley (SD) rats [12]. Hence, it needs frequent dosing to maintain a sufficient drug concentration in clinical trials, which affects the living quality of clinical patients.

In order to extend the in vivo half-life of peptides, various pharmaceutical technologies have been developed, such as fusion protein formation, chemical modification and glycosylation modification, among which PEG modification and fusion protein technology represented by Fc-fusion are the most prominent [13,14]. Both methods are applied in many successfully listed drugs. Compared with chemical modification, fusion protein technology greatly prolongs the half-life of drugs while obtaining more uniform product, higher yield and easier purification [15]. Moreover, the fusion protein is a bi-functional molecule that can lead to new biological functions to effector molecules. For instance, the FcRn receptor-mediated recycling mechanism can further extend the in vivo half-life of the fusion proteins, which has a wider application prospect than chemical modification methods.

Currently, Fc fusion is the most popular and fastest-growing protein fusion technology [16]. It uses the crystalline fragment (Fc) segment of immunoglobulin (IgG) as a molecular chaperone to fuse functional proteins by means of molecular biology, which maintains the activity of functional proteins and keeps the long half-life of immunoglobulins as well [17]. Its fusion targets involve receptor domains, ligands, antibody fragments and peptides and it has evolved as a reliable drug development instrument [18]. Fc-fusion proteins greatly increase the molecular weight of proteins and peptides and reduce the glomerular filtration rate [19]. FcRn-mediated recycling mechanisms successfully avoid protein degradation and effectively increase half-life [20,21]. In addition, human Fc fragments reduce the immunogenicity of the fusion protein, thereby elimination of drugs by the immune system is prevented.

The half-life of the Fc fusion protein has been greatly improved in drugs listed in the market [22,23]. As it has been reported, the half-life of ahatacept is up to 13.1 days and that of aldfacept is 12 days [24]. Drugs that are already on the market, such as etanercept, not only retain the biological activity of the fusion target but also keep Fc antibody activity. The efficacy of the drug can be compared to that of the cloned antibody and the market responds strongly after its listing. In 2010, Seap’s global sales reached US $7.3 billion, surpassing the monoclonal antibody drugs such as rituximab and infliximab [25].

Although there are many successful examples of the utilization of Fc fusion protein technology for polypeptides, the development of HM-3-Fc is still the first case. On the basis of maintaining the efficacy of HM-3, HM-3-Fc will further increase the half-life of HM-3 in vivo, while using the Fc fragment as a purification label to substantially streamline the production process. HM-3-Fc is very promising in drug discovery. Our study mainly focuses on the physiochemical characteristics, in vitro and in vivo pharmacodynamics and demonstrates the drug properties of HM-3-Fc.

## 2. Results

### 2.1. Physiochemical Characteristics of HM-3-Fc

**Figure 1:**
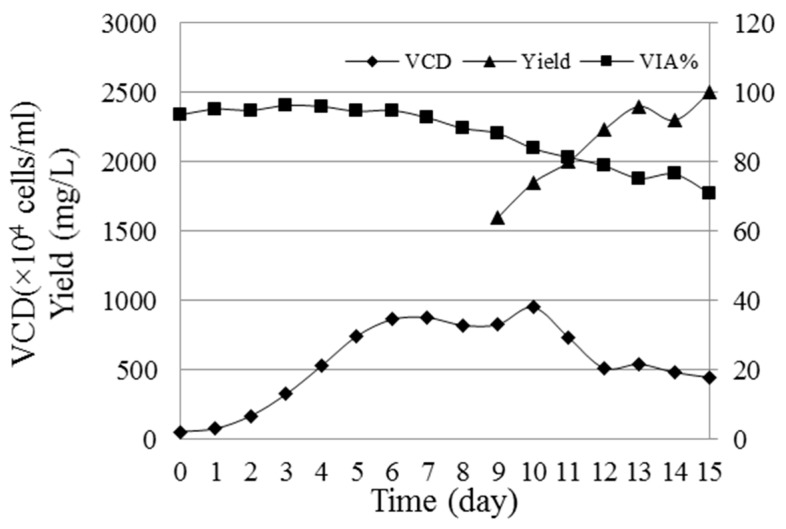
The viability (VIA%), viable cell density (VCD) and yield of HM-3-Fc cells cultured for 15 days. Sterilely taken cells from bioreactor were stained by 0.4% trypan blue and then counted with a Countstar IC-1000 cell counter. The results of viable cells are summarized; VIA% = number of viable cells/total cell number × 100%. The protein expression amount was detected by size exclusion chromatography-high pressure liquid chromatography (SEC-HPLC) method.

Full gene of HM-3-Fc contains a signal peptide, IgG4-Fc, 15-amino-acid GGGGS Linker and HM-3 by full gene synthesis technology and this full gene was inserted in a pCHOGUN vector. The vector was transfected to Gene Pulser X cells using the X cell electroporator. Clones originating from a single cell were isolated by limiting dilution. Stable cell line was obtained by glutamine deficiency cultivation and by adding 25 μM methionine sulfoximine (MSX). The HM-3-Fc cells were cultured and amplified to a final 5 L bioreactor for Fed-batch cultivation. During 15 days of Fed-batch cultivation, the cell density (VCD) reached a maximum of 8.7 × 10^6^ cells/mL on the 6th day and the cell viability (VIA) remained 70.95% at the end of culture, while the yield of HM-3-Fc was 2.5 g/L (Figure 1).

**Figure 2:**
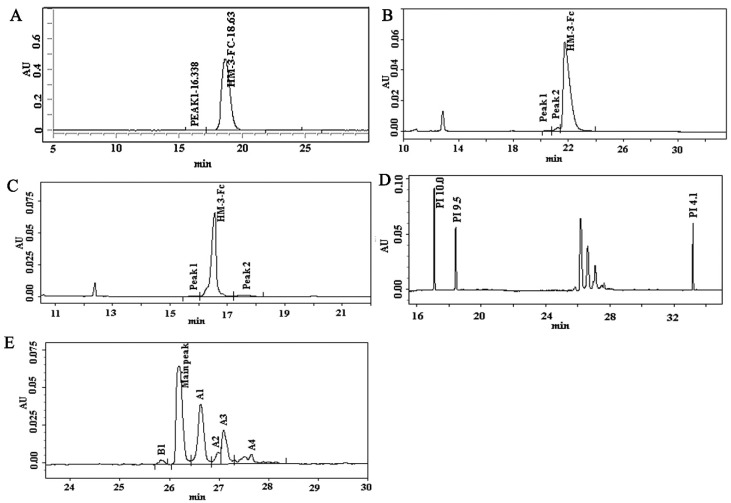
Physiochemical characteristics of HM-3-Fc. (1) Purity analysis of HM-3-Fc (**A**). Columns: TOSOH, TSKgel G3000SWxL PN: 008543, Mobile Phase: 0.4 M NaCl 0.03 M Na_2_HPO_4_ 0.02 M NaH_2_PO_4_ Solution with 10% Acetonitrile. Isocratic elution, Flow: 0.5 mL/min, Temperature: 25 °C, Detection fluorescence (FL): 214 nm, Injected volume: 10 μL. (2) CE-SDS analysis of HM-3-Fc. Non-reduced capillary electrophoresis (CE) analysis (**B**) and reduced CE-SDS analysis (**C**). Sample concentration: 1 mg/mL heated in water bath at 70 °C for 10 min. The sample was injected to the anode with reverse polarity using −5 kV for 20 s. Separation voltage was −15 kV with reverse polarity during the 30 min separation. Detection wavelength was 214 nm. (3) cIEF analysis of HM-3-Fc. Holograph of cIEF profile (**D**) and amplified part of cIEF graph (**E**). B1 = basic peak 1, A1–A4 = acidic peaks 1–5. The samples were detected by Beckman MDQ capillary electrophoresis system using a neutral coated capillary. Inject sample for 99.0 s at 20 psi. Focusing: 15 min at 25 kV under normal polarity. Mobilization: 30 min at 30 kV under normal polarity. Detection wavelength was 280 nm.

During HM-3-Fc purification, the culture supernatant was first loaded into a Mabselect SuRe affinity column to capture the target protein (Supplementary Figure S1), then the fraction pool containing HM-3-Fc protein was loaded into a Superdex 200 column for further purification of the target protein (Supplementary Figure S2). The purified HM-3 fusion protein was analyzed by size-exclusion high performance liquid chromatography (SEC-HPLC), capillary electrophoresis with sodium dodecylsulfate (CE-SDS), capillary isoelectric focusing (cIEF) and other methods for quality control. The concentration of HM-3-Fc fusion protein was 16.42 mg/mL and the purity result of the SEC-HPLC assay was 99.42% (Figure 2A). The high purity was consistent with a single peak presence of HM-3-Fc on the non-reduced and reduced CE-SDS results (Figure 2B,C). The range of isoelectric point was from 5.971 to 6.787 and the main peak was 6.681, which was consistent with the theoretical value (Figure 2D). In this part of work, high performance liquid chromatography, capillary electrophoresis with sodium dodecylsulfate and capillary isoelectric focusing technique were used instead of traditional gel electrophoresis, since these up-to-date techniques are more accurate and sensitive.

### 2.2. Target Affinity Evaluation

**Figure 3:**
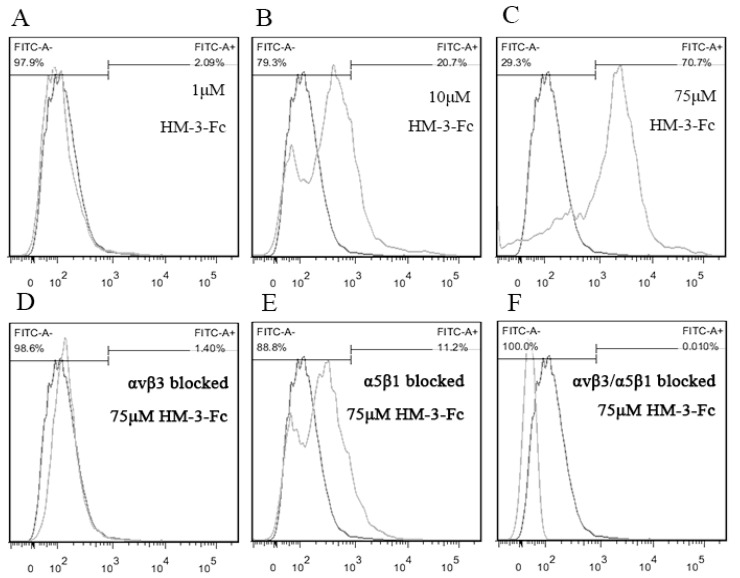
Specific and high affinity binding of HM-3-Fc with integrin αvβ3 and α5β1. A flow cytometry method was setup with FITC-protein A (FITC-A) as a probe that binds the Fc part of HM-3-Fc. The dark line indicated the fluorescence signal for untreated synovial cells and it was used to define the threshold of positive fluorescence signal. The light line indicated the fluorescence signal for HM-3-Fc and FITC-A treated synovial cells. (**A**–**C**) The binding rates of synovial cells after incubation with 1, 10 or 75 μM HM-3-Fc before incubated with FITC-A. (**D**–**F**) The binding rates of synovial cells that were blocked with anti-αvβ3, anti-α5β1, or their combination.

To confirm the specific and high affinity binding of HM-3-Fc with integrin αvβ3 and α5β1, a flow cytometry detection of HM-3-Fc coated synovial cells was performed with FITC-protein A as a probe. HM-3-Fc binding rate of untreated mouse synovial cells and the corresponding fluorescence signal increased with increasing concentrations of HM-3-Fc (Figure 3A–C). Under the treatment with 75 μmol/L HM-3-Fc, the binding rate was 70.7%, in other words, 70.7% cells showed positive fluorescence signal (Figure 3C). To confirm that HM-3-Fc interacted with integrin αvβ3 and α5β1, antibodies specific for integrin αvβ3 or α5β1 or their combination were used to incubate the synovial cells before their incubation with 75 μmol/L HM-3-Fc. As shown in Figure 3D–F, when mouse synovial cells were blocked with an anti-αvβ3 antibody, the binding rate of HM-3-Fc was 1.40% (Figure 3D). When the cells were blocked with an anti-α5β1 antibody, the binding rate was 11.2% (Figure 3E). And after αvβ3 and α5β1 were blocked simultaneously, the binding rate was reduced to 0.01% (Figure 3F). The experiments were repeated twice. These results confirmed that HM-3-Fc interacted with integrin αvβ3 and α5β1 expressed on synovial cell surface.

### 2.3. The Effects of HM-3-Fc In Vitro

#### 2.3.1. The Effect of HM-3-Fc on Splenic Lymphocytes Viability

**Figure 4:**
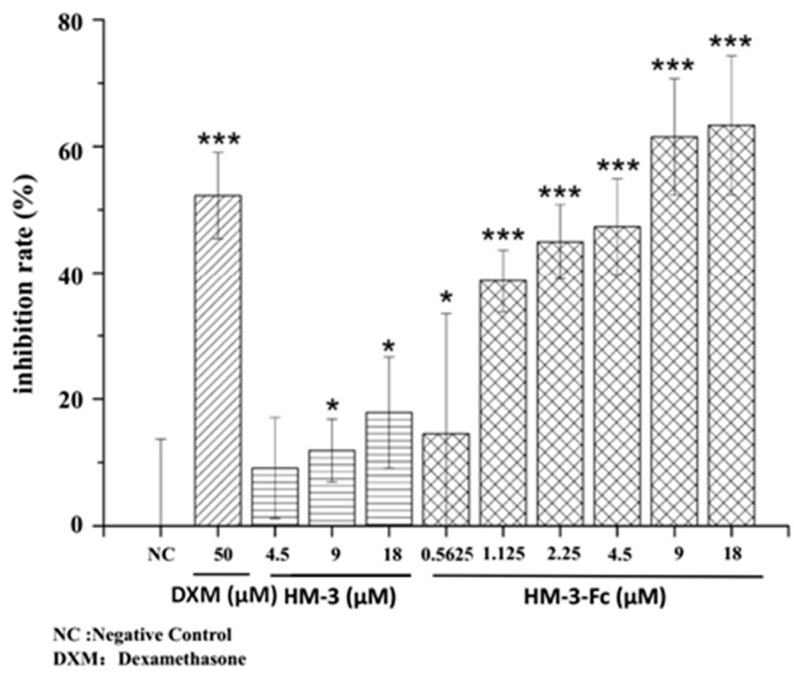
The effects of HM-3-Fc on splenic lymphocytes viability. Cells were stimulated with ConA, based on which 50 μmol/L Dexamethasone (DXM) was used as a positive control drug. In the other groups, cells were incubated with different concentrations of HM-3 or HM-3-Fc as indicated in the Figure. In this study, proliferation inhibition rate (PI) was used to express the inhibitory effect of HM-3-Fc on the proliferation of modeled splenic lymphocytes. The formula is as follows: PI = 1 − A_test_/A_control_100%. A_test_ is the absorbance of the drug group. A_control_ is the absorbance of the negative control group. Results were presented as mean ± SD (*n* = 6). * *p* < 0.05 and *** *p* < 0.001.

To evaluate the in vitro anti-inflammatory activity of HM-3-Fc, its effect on the splenic lymphocyte viability was analyzed by the 3-(4,5-Dimethylthiazol-2-yl)-2,5-diphenyltetrazolium bromide (MTT) assay. Briefly, the cells were incubated with different drugs for 48 h before MTT incubation and optical density (OD) measurement. We can see from Figure 4 that the inhibition rate of positive control Dexamethasone (DXM, 50 μmol/L) was 52.3 ± 6.8%. Peptide HM-3 showed a dose-dependent inhibition of splenic lymphocyte viability at 18 μmol/L and the inhibition rate of HM-3 was 18%. HM-3-Fc also showed a dose-dependent inhibition of splenic lymphocyte viability and the inhibition rate of HM-3-Fc was higher than that of HM-3 at the corresponding dose. At 18 μmol/L, the inhibition rate of HM-3-Fc was 63.3%, which is even higher than the positive control drug (Figure 4). Photographs of the splenocytes before or after DXM, HM-3 or HM-3-Fc treatments confirmed the MTT results (Supplementary Figure S3). There were abundant cells in the well that was incubated with ConA without drug treatment, whereas in the DXM treated well, the cell number was much lower. In HM-3 or HM-3-Fc treated wells, the appearing cell number also decreased (Supplementary Figure S3). 50 μmol/L DXM was selected as a positive reagent based on its significant inhibition on splenic lymphocyte viability at this concentration (Supplementary Figure S4). The results indicated that HM-3-Fc significantly inhibited the viability of splenic lymphocytes isolated from the mice stimulated by Concanavalin A (ConA), which was superior to that of HM-3.

#### 2.3.2. Down Regulation of TNF-α Expression by HM-3-Fc in Macrophage U937

**Figure 5:**
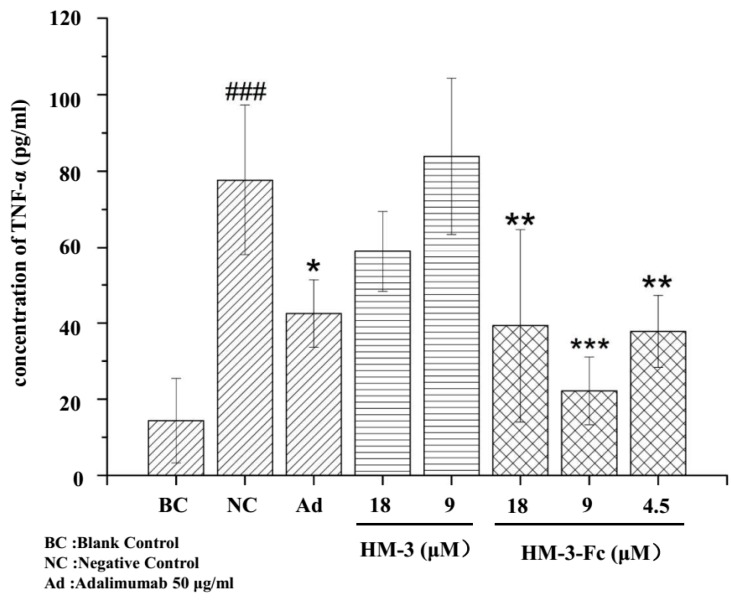
The effects of HM-3 and HM-3-Fc on the expression of TNF-α by U937 macrophages. Cells without LPS induction were used as a normal control. The other cells were inducted with LPS and the cells without drug treatment were used as a negative control. The other cells were incubated with HM-3 or HM-3-Fc at indicated concentrations, with 50 μg/mL Adalimumab as a positive control drug. Results were presented as mean ± SD (*n* = 3). ### *p* < 0.001 vs. Blank Control. * *p* < 0.05, ** *p* < 0.01 and *** *p* < 0.001 vs. Negative Control.

5 × 10^4^ U937 cells (10 μL) were added to each well of a 96 well plate and were stimulated with 90 μL LPS (1 μg/mL). Different concentrations of HM-3 and HM-3-Fc were incubated at the same time with 50 μg/mL Adalimumad as a positive control drug. The concentration of TNF-α in the supernatant was measured. We can see from Figure 5 that after LPS stimulation, the concentration of TNF-α in the negative control was significantly higher than that in the normal control (without LPS stimulation). Peptide HM-3 did not show a significant inhibition of TNF-α secretion. HM-3-Fc could significantly inhibit the TNF-α expression in the medium of LPS-stimulated macrophages and 9 μM HM-3-Fc showed the strongest inhibitory effect. To exclude the possibility that HM-3-Fc decreased the viability of U937 cells, MTT method was performed, to evaluate the cytotoxic effect of HM-3-Fc on macrophage U937 cells. After drug treatment for 48 h, cell supernatant was collected for TNF-α detection and the same volume of fresh culture medium was added. Routine MTT procedure was followed and it was found that Adalimumab, HM-3 or HM-3-Fc did not result in a significant change in U937 viability (Supplementary Figure S5).

### 2.4. HM-3-Fc Inhibits Angiogenesis In Vivo

**Figure 6:**
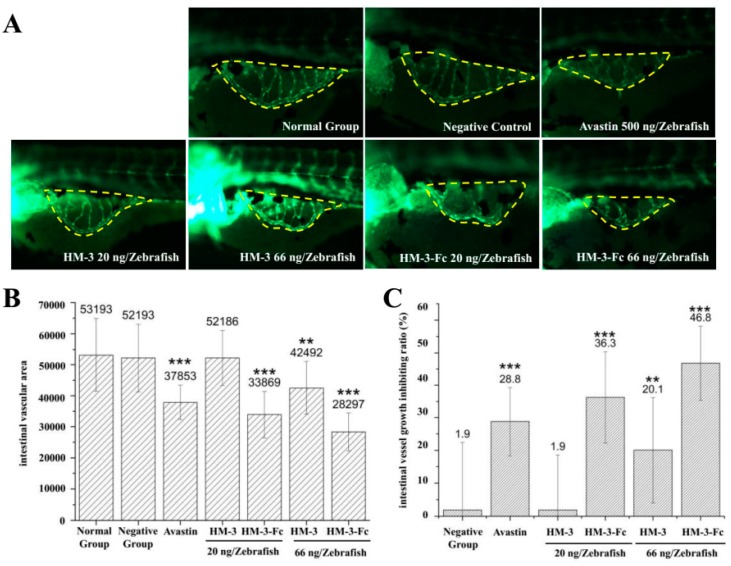
Inhibitory effect of HM-3-Fc on angiogenesis in zebrafish. Zebrafish were subcutaneously injected with HM-3 or HM-3-Fc and also with Avastin as a positive control. After 24 h, intestinal vascular area was visualized and measured and then angiogenesis inhibition rates were calculated based on the vascular area of each group. (**A**) Typical photos of zebrafish intestinal vascular area in different groups (150×). (**B**) The intestinal vascular areas of zebrafish in each group were visualized and measured by Nikon NIS-Elements D 3.10. And the areas of intestinal blood vessels in the experiment were calculated (S). (**C**) Angiogenesis inhibition rate = (1 − S(Normal Group)/S (Experiment Group)) × 100%. Results were presented as mean ± SD (*n* = 12). ** *p* < 0.01, *** *p* < 0.001.

Zebrafish were subcutaneously injected with HM-3 or HM-3-Fc and with Avastin as a positive control. Zebrafish that were only injected with buffer were used as a negative control and those without any treatment were normal group. As shown in Figure 6, the positive control (500 ng Avastin per zebrafish) showed a 29% inhibition of aniognenesis compared with that of a normal control, which demonstrated that Avastin had a strong effect of anti-angiogenesis. 66 ng HM-3-Fc per zebrafish treatment resulted in the intestinal vascular area of 28297 (Figure 6B) and its inhibition rate of angiogenesis was 47% (Figure 6C). The results indicated that HM-3-Fc had a strong anti-angiogenesis property at a dose of 66 ng per zebrafish. 66 ng HM-3 per zebrafish inhibited angiogenesis by 20%. We concluded that HM-3-Fc had a stronger anti-angiogenesis effect, which was better than that of Avastin and HM-3.

### 2.5. The Protective Effect of HM-3-Fc in a Collagen-Induced Arthritis (CIA) Model

#### 2.5.1. The Effects of HM-3-Fc on Mice Paw

A collagen-induced arthritis model in mice was established by an immunization via intradermal injection of 50 μL emulsion of bull collagen type II and Complete Freund’s adjuvant (CFA) on day 0 and a subsequent injection of incomplete Freund’s adjuvant (IFA) on day 21. On the 30th day of the experiments, the CIA model mice were randomized into 7 groups and a group of normal mice without immunizations and drug treatments were used as a normal control. The strategy of mice treatments was in Table 1.

The dynamic change of paw thickness, tarsus width and paw parameter with days of drug treatments were shown in Supplementary Figure S6. In general, mice in G3 showed significant decrease of these parameters compared with the model mice in G2 at the earliest time point. Mice in G5 (50 mg/kg), G6 (25 mg/kg) and G7 (12.5 mg/kg) that were subcutaneously administered with HM-3-Fc once every 5 days also showed significant decrease of these parameters and lower dose of HM-3-Fc treatment resulted in a longer time until when there appeared significant difference with the mice in G2. Mice in G8 (25 mg/kg, once every 7 days) showed significant decrease at the same time with mice in G7. And mice in G4 always showed significant difference at a later time point than the mice in G3 and G5 to G8. Average paw perimeters of mice in all groups on day 60 were shown in Table 2.

The results of paw thickness, tarsus width and paw perimeter indicated that HM-3-Fc treated mice had significant differences compared with the model control group mice (Table 2). Subcutaneous injections of 50 or 25 mg/kg HM-3-Fc once every 5 days and subcutaneous injections of 25 mg/kg once every 7 days had a better protecting effect than Adalimumab or HM-3 treatment.

#### 2.5.2. Arthritis Grading of CIA Mice after HM-3-Fc Treatments

**Figure 7:**
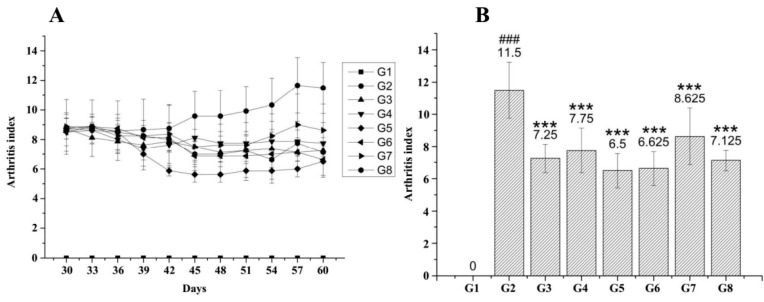
HM-3-Fc treatments decreased arthritis grading of CIA mice. Mice were immunized with an emulsion of bull collagen type II and Complete Freund’s adjuvant (CFA) on day 0 and subsequently with an injection of incomplete Freund’s adjuvant (IFA) on day 21. On the 30th day of the experiments, mice were randomized in different groups for treatments with different drugs. Arthritis grading (**A**) of mice in each group was measured every three days until the 60th day. Comparison of arthritis index of the mice in each group was shown in panel (**B**). For G2, *n* = 12; for G3–G8, *n* = 8. ### *p* < 0.001 vs. G1. *** *p* < 0.001 vs. G2.

Dynamic change of arthritis grading of CIA mice in each group was shown in Figure 7. Severe arthritis symptoms occurred in the model control group and drug treatments resulted in reduction of arthritis grading. On Day 60, the mice arthritis grading of G3, G4, G5, G6, G7 or G8 group was 7.6 ± 0.9, 7.8 ± 1.4, 6.5 ± 1.1, 6.6 ± 1.1, 8.6 ± 1.8 or 7.1 ± 0.6, respectively and they showed significant differences from that of model control group mice (11.5 ± 1.7, ** *p* < 0.01). The results confirmed that subcutaneous injections of 50 or 25 mg/kg HM-3-Fc once every 5 days and subcutaneous injections of 25 mg/kg once every 7 days had a better protective effect than Adalimumab or HM-3 treatment (Figure 7).

The results of body weight change (Supplementary Table S1) and in vitro cytotoxic experiment (Supplementary Figure S5) showed that none of the treatment group had obvious cytotoxic effect.

The results above suggested that different doses and frequencies of HM-3-Fc treatments had different efficacies on CIA model mice. G3, G4, G5, G6, G7 and G8 groups showed better drug efficacy. Paw width, tarsus width, arthritis grading, spleen weight and paw weight of these drug-treated groups showed significant difference compared with the model control group. Subcutaneous injections of 50 mg/kg (G5) or 25 mg/kg (G6) HM-3-Fc once every 5 days and subcutaneous injections of 25 mg/kg once every 7 days (G8) showed better efficacy than positive control group (G3) and HM-3 treatment group (G4). According to the results of paw thickness, tarsus width, paw perimeter and arthritis grading, we figured out that during and at the end of the treatment, subcutaneous injections of 50 mg/kg (G5) or 25 mg/kg (G6) HM-3-Fc once every 5 days and subcutaneous injections of 25 mg/kg once every 7 days (G8) showed better efficacy than G3 (Adalimumab). Subcutaneous injections of 12.5 mg/kg HM-3-Fc once every 5 days (G7) showed less efficacy than G3 and G4 but they showed significant difference with the model control group (G2).

### 2.6. Preliminary Pharmacokinetic Study of HM-3-Fc in Cynomolgus

**Figure 8:**
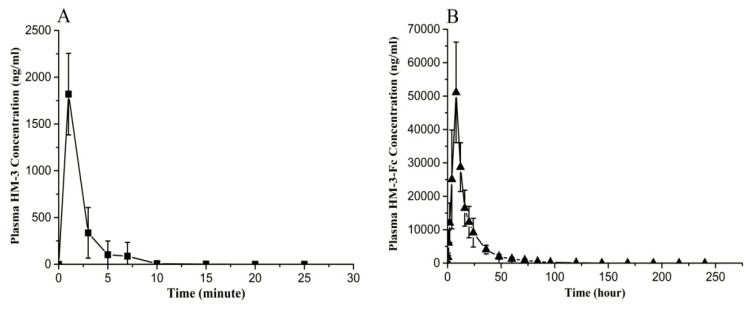
Serum drug concentration versus time curves of HM-3 and HM-3-Fc after subcutaneous injection in cynomolgus. (**A**) Serum drug concentration versus time curve of HM-3 after s.c. administration of 0.5 mg/kg HM-3 in cynomolgus. (**B**) Serum drug concentration versus time curve of HM-3-Fc after s.c. administration of 5.77 mg/kg HM-3-Fc in cynomolgus. Data were shown as mean ± SEM (standard error of mean).

We conducted preliminary pharmacokinetic study of HM-3-Fc in comparison with HM-3 in cynomolgus. After a single subcutaneous injection of 0.5 mg/kg HM-3, the in vivo HM-3 concentration increased immediately to the maximum concentration (1819.52 ng/mL) and then decreased to the base value within 10 min, with a half-life of 1.32 min (Table 3 and Figure 8A). For HM-3-Fc, after a single subcutaneous injection of 5.77 mg/kg HM-3-Fc, the in vivo HM-3-Fc concentration increased gradually to the maximum concentration of 56112.3 ng/mL and gradually decrease thereafter. The in vivo half-life of HM-3-Fc was 15.24 h. The concentration of HM-3-Fc decreased to baseline level after 5 days (Table 3 and Figure 8B).

## 3. Discussion

In this study, we evaluated the efficacy of HM-3-Fc fusion protein in RA treatment. The ConA-stimulated splenocyte viability and TNF-α secretion from macrophage experiments confirmed that HM-3-Fc had an anti-inflammatory activity which may help in alleviating RA syndromes. HM-3-Fc also inhibited angiogenesis in zebrafish. In vivo pharmacodynamics studies showed that subcutaneous injections of 50 or 25 mg/kg HM-3-Fc once every 5 days alleviated RA syndromes better than the positive drugs adalimumab and HM-3. Most times the fusion of peptide and Fc reduced the efficacy of the active peptide in vivo [26]; however, in our research, HM-3-Fc showed better efficacy than HM-3 in the improvement of arthritis index (Figure 8), TNF-α expression by macrophages (Figure 5) and CD31 expression in the immunohistochemical analysis (Supplementary Figure S7C). Preliminary pharmacokinetic study in cynomolgus found that the in vivo half-life of HM-3-Fc was 15.24 h, which is much longer than the half-life of 1.32 min in cynomolgus. 

There are three major mechanisms of HM-3 effect: inhibition of angiogenesis, suppression of chemotaxis of immune cells and reduction of inflammatory factors [27]. At present, HM-3 related peptides have not yet been approved for marketing. In cancer treatment researches, HM-3 has entered phase I clinical trial but no drugs have entered the clinical stage in the treatment of rheumatoid arthritis. As RA is a chronic disease that requires long-term treatment, it is imperative to develop long-acting macromolecular drugs based on HM-3. Our previous studies demonstrated that HM-3 alleviated rheumatoid arthritis syndromes by inhibiting the migration of vascular endothelial cells, inhibiting T cell division and inhibiting the expression levels of TNF-α and VEGF by LPS-activated macrophages. The efficacy of HM-3 in RA treatment was demonstrated by the in vivo pharmacodynamics study in adjuvant-induced arthritis (AIA) and CIA rats [26].

Our HM-3-Fc fusion protein was designed for extension of in vivo half-life of HM-3. The fusion protein was created by fusing the Fc-domain of modified human IgG4 and HM-3 with a 15-amino-acid GS as a linker. HM-3-Fc significantly prolonged the half-life of HM-3 without losing HM-3 bio-activity. In order to evaluate the anti-RA activity of HM-3-Fc in vivo, we used a CIA model mice and we found that subcutaneous injections of 25 mg/kg HM-3-Fc once every 7 days showed the same anti-RA activity as administration of the same dose once every 5 days and the key indicators were better than the positive control drug Adalimumab (Figure 7, Supplementary Figure S6, Table 1, Supplementary Table S1). If the frequency of administration was systematically optimized and increased to 7 days, the half-life in the human body may be further extended to 15 days. Meanwhile pharmacokinetic studies in plasma of cynomolgus showed that after a single dose treatment, the HM-3-Fc group exhibited a dramatically longer half-life, higher Cmax and larger AUC0−240 h than the HM-3 group. The half-life of HM-3-Fc in cynomolgus was extended to 15 h, which was almost 700 times than that of HM-3 polypeptides. According to the experimental data, we supposed the frequency of HM-3-Fc administration could be extended to once every 15 days. Compared with HM-3 that need an administration of twice daily, HM-3-Fc can significantly improve life quality and compliance of RA patients.

With the maturation of biotechnology and peptide synthesis technology, more and more peptide drugs have been developed and applied in clinical practice. Due to the wide range of indications, high safety and significant efficacy, peptide drugs have been widely used in the prevention, diagnosis and treatment of diseases such as cancer, hepatitis and diabetes, which have broad prospects for development [11,28]. However, polypeptide drugs also have unavoidable limitations, including their easy degradation by proteases, short half-life and instability [29]. In previous study, long-acting Fc-fusion proteins were constructed by Fc-fusion technology and preliminary explorations were performed to ensure the retention of the biological and pharmacological activities of the original polypeptide drugs. After significant increase of the peptide half-life, research for new applications and indications and technology platform for new drug development as macromolecular proteins are needed.

Up to now, the present study has completed preliminary process for fusion protein preparation and product quality control. In vivo and in vitro experiments have proved that HM-3-Fc effectively alleviated RA syndromes by inhibiting angiogenesis and anti-inflammatory effects. The fusion protein technology makes it possible to significantly prolong the half-life of HM-3. This study also demonstrated the target specificity of HM-3-Fc. To further evaluate the anti-RA properties of HM-3-Fc, more works are needed. First, the efficacy of HM-3-Fc in cynomolgus needs to be studied to provide theoretical support for the later toxicological studies and the selection of clinical doses. Secondly, systematic safety evaluation of HM-3-Fc, including acute toxicity, long-term toxicity and immunogenicity is needed to fulfil preclinical research data for investigational new drug application.

## 4. Materials and Methods

### 4.1. The Preparation of HM-3-Fc

#### 4.1.1. Screening of HM-3-Fc Stable Cell Line

GS-CHO-K1 cell line and the corresponding pCHOGUN vector were supplied by Horizon (Horizon Discovery Ltd., Cambridge, UK). The full gene containing a signal peptide, IgG4-Fc, a 15-amino-acid GGGGS Linker and HM-3 was obtained by full gene synthesis technology and this full gene was incorporated into pCHOGUN vector.

The vector was transfected to host cells using the X cell electroporator (Bio-rad, Hercules, CA, USA, Gene Pulser Xcell). Clones originating from a single cell were isolated by limiting dilution. The stable cell line was obtained by glutamine deficiency cultivation and by adding 25 μM MSX.

#### 4.1.2. Cultivation of HM-3-Fc Fusion Protein Producing Cell Line

The cells stably secreting HM-3-Fc were resuscitated in a 125 mL flask. After amplifying cultivations, the cells were finally seeded in a 5 L bioreactor BIOSTAT^®^ B plus (Sartorius, Göttingen, Germany) for Fed-batch cultivation. The initial volume was 2.5 L. The medium and additive were Excell Advanced^TM^ CHO Fed batch Media and Excell Advanced^TM^ Feed 1 (Sigma, St. Louis, MO, USA), respectively.

In the process of cell culture, we mainly focused on cell density, viability, glucose and lactic acid metabolism (data not shown in this paper), protein expression and so forth. Countstar IC-1000 was used to track cell growth, mainly included living cell density and viability (cell viability = viable cell density/total cell density × 100%); at the same time, SEC-HPLC was used to detect the protein amount in the fermentation broth and the detection method is described in Section 4.1.4.

#### 4.1.3. Purification of HM-3-Fc Fusion Protein

The cells were removed by centrifugation. The culture supernatant was first loaded into Mabselect SuRe affinity column (GE) to capture the target protein. After that, the fraction pool containing HM-3-Fc was loaded into Superdex 200 column (GE) for further purification of the target protein. In details, 6.23 L culture supernatant was filtered through a 0.45 um filter and was loaded onto a Protein A affinity column (XK50/20, 294 mL, 15 cm) with a flow rate of 20 mL/min at 4 °C. The affinity column was sanitized with 0.2 M NaOH and equilibrated with 20 mM phosphate buffer containing 0.15 M NaCl (pH 7.0). After sample loading, the column was washed with 5 column volume of washing buffer 1 (20 mM phosphate buffer containing 0.15 M NaCl, pH 7.0), then with 5 column volume of washing buffer 2 (20 mM phosphate buffer containing 1 M NaCl, pH 7.0) and finally with 5 column volume of washing buffer 3 (50 mM citric/citrate containing 0.15 M NaCl, pH 3.0). HM-3-Fc was eluted by 50 mM Citric/Citrate, 0.15 M NaCl, pH 3.0. A single peak with high protein concentration was obtained after elution with a shoulder peak. The affinity column was cleaned with 0.2 M NaCl. To remove the impurity in the shoulder peak, the elution fraction from the affinity column was further purified by a size exclusion column. 65 mL Protein A column elution sample was loaded onto Sephadex 200 size exclusion chromatography (XK50/100, Volume 1531 mL, Height 78 cm) with a flow rate of 8 mL/min. The column was sanitized with 0.2 M NaCl and equilibrated with 20 mM phosphate buffer containing 0.15 M NaCl (pH 7.0). HM-3-Fc was eluted by 50 mM Citric/Citrate, 0.15 M NaCl, pH 3.0. A single peak with high protein concentration was obtained after elution. The purity was up to 99% in HPLC detection. Finally, the protein solution was concentrated to a proper concentration according to its OD value, followed by the sterile filter packing of the sample.

#### 4.1.4. Analysis of Purified HM-3-Fc Fusion Protein

The purified protein concentration was analyzed by UV spectrophotometer (GE, Ultrospec3000 pro, Little Chalfont, Buckinghamshire, UK). To select the proper wavelength range, scan interval was selected within 190–400 nm. The protein sample was processed by centrifugation at 12000 rpm for 10 min. Then the protein purity was detected by SEC-HPLC using TSK gelG3000WxL (7.8 × 300 nm) (Tosoh Corporation, Tokyo, Japan). Mobile Phase: 0.4 M NaCl 0.03 M Na_2_HPO_4_ 0.02 M NaH_2_PO_4_ solution with 10% Acetonitrile. Isocratic elution, Flow: 0.5 mL/min, Temperature: 25 °C, Detection FL: 214 nm, Injected volume: 10 μL. After desalinization, the reducing and non-reducing samples were processed by β-mercaptoethanol and Iodoacetamide, respectively. The protein purity was detected by capillary electrophoresis apparatus (Beckman, city, country, P/ACE MDQ) and PN 144733 UV detector (Beckman, Miami, FL, USA, PN 144733). Sample concentration: 1 mg/mL heated in water bath at 70 °C for 10 min. The sample was injected to the anode with reverse polarity using −5 kV for 20 s. Separation voltage was −15 kV with reverse polarity during the 30 min separation. Detection wavelength was 214 nm. At the same time, the protein isoelectric point was detected by cIEF to track the quality of the product. The samples were detected by Beckman MDQ capillary electrophoresis system using a neutral coated capillary. Inject sample for 99.0 s at 20 psi. Focusing: 15 min at 25 kV under normal polarity. Mobilization: 30 min at 30 kV under normal polarity. Detection wavelength was 280 nm. 

### 4.2. Target Affinity Study

Mouse synovial cells were obtained after digestion and centrifugation. Cells were blocked with 1% BSA for half an hour at 4 °C and were washed with PBS twice. Three unblocked groups were incubated with 1, 10 or 75 μM HM-3-Fc at the ratio of 1:1 for 1 h at 4 °C. Cells in the three antibody-blocked groups were incubated with the blocking antibody for half an hour at 4 °C. After washing with PBS twice, the cells were centrifuged and then re-suspended with PBS; then the re-suspended cells were incubated with 75 μM HM-3-Fc at the ratio of 1:1 for 1 h at 4 °C. After complete incubation, following washing with PBS twice and centrifuge, the cells were re-suspended with 200 μL PBS. The fluorescence signal of the cells in each condition was analyzed with a flow cytometer (Miltenyi, MACSQuant, Bergisch Gladbach, Germany).

### 4.3. The Effects of HM-3-Fc In Vitro

#### 4.3.1. Splenic Lymphoproliferative Study

Mice were killed after orbital bleeding and then were immersed into 75% alcohol immediately for 5 to 10 min. The spleen was freshly isolated and put into PBS in laminar flow bench. The isolated spleen was put onto a sterile cell sieve and grinded with a syringe head, during which period PBS was continuously added. The lapping liquid was centrifuged at 1000 rpm for 5 min and the collected cells were washed with Tris-HCl solution for three times and then with the media once. The splenocytes were re-suspended in media and were counted after staining with Trypan Blue to make sure that the cell viability was up to 95%. The cells were diluted to a final concentration of 2 × 10^6^ cells/mL and were cultured in flat-bottom 96-well plates (100 μL per well) in presence of 0.5 μg/mL/well ConA. At the same time, 90 μL of the following drugs were added and there were 11 groups of 6 repeating wells: the control group containing 100 μL media without ConA, Dexamethasone (50 μmol/L), HM-3 (4.5, 9, 18 μmol/L), HM-3-Fc (0.5625, 1.125, 2.25, 4.5, 9, 18 μmol/L). The 96-well plate was incubated in the incubator (37 °C, 5% CO_2_). After this specified treatment for 48 h, 20 μL MTT (5 mg/mL) was added in each well and the plate was incubated in the incubator for another 4 h. The media solution in the plate was discarded and 100 μL DMSO was added to each well and mixed gently. The conversion of MTT to formazan in metabolically viable cells was spectrophotometrically measured at 570 nm, with 630 nm as a reference wavelength. The effect of Dexamethasome on the viability of splenic lymphocytes was performed to choose an optimal Dexamethasome concentration. Fresh isolated splenic lymphocytes were stimulated with 0.5 μg/mL ConA in absence or presence of 0.8, 4, 20 or 100 μg/mL Dexamethasome. Inhibitory effect of Dexamethasome on splenic lymphocyte viability was evaluated in comparison with ConA stimulated sample.

#### 4.3.2. The Inhibition of TNF-α Expression in U937 Macrophage

U937 macrophages were cultured in RPMl-1640 media containing 10% FBS and Penicillin-Streptomycin in the incubator (37 °C, 5% CO_2_). The cells were harvested and suspended to a final density of 5 × 10^5^ cells/mL. 100μL cells per well were added to a 96-well plate to be incubated overnight. On the following day, LPS (1 μg/mL, 90 μL) was added to activate U937 macrophages. Simultaneously, different drugs were added as a treatment: blank control group (100 μL media without LPS); negative control group (90 μL LPS); Adalimumab (50 μmol/L); HM-3 (9, 18 μmol/L); HM-3-Fc (4.5, 9, 18 μmol/L). After 48 h of treatment, TNF-α concentration in the supernatant was tested by ELISA. After drug treatment for 48 h, cell supernatant was collected for TNF-α detection and the same volume of fresh culture medium was added. Then, 20 μL MTT (5 mg/mL) was added in each well and the plate was incubated in the incubator for another 4 h. The media solution in the plate was discarded and 100 μL DMSO was added to each well and was mixed gently. The conversion of MTT to formazan in metabolically viable cells was spectrophotometrically measured at 570 nm with 630 nm as a reference wavelength.

### 4.4. HM-3-Fc Inhibits Angiogenesis In Vivo

Transgenic vascular fluorescent zebrafish were randomized into 6-well plates, with 30 zebrafish per well. They were each treated with HM-3 (20 ng/zebrafish or 66 ng/zebrafish), HM-3-Fc (20 ng/zebrafish or 66 ng/zebrafish), positive control Avastin (500 ng/zebrafish), buffer control group (buffer only) and normal control group (no treatment). The drugs were injected subcutaneously with amount of 20 ng/zebrafish. After treated for 24 h, 10 zebrafish were randomly selected from each group and their SIVs were observed under the fluorescence microscope. Pictures were obtained and analyzed with Nikon NIS-Elements D 3.10 advanced image processing software in order to calculate the areas of SIVs of experiment groups.

### 4.5. In Vivo Study

#### 4.5.1. The Induction of CIA

Type II collagen from bull was diluted to 4 mg/mL and then it was emulsified with an equal volume of Complete Freund’s adjuvant (CFA). Except for the normal control group, Balb/c mice were immunized intradermally at the base of the tail with 50 μL emulsion. 21 days later, the mice were re-immunized with the same amount of emulsion which contains incomplete Freund’s adjuvant (IFA). Around the 29th day from the first immunity, inflammation symptoms such as swelling occurred which indicated the success of the induction.

#### 4.5.2. Drug Treatments

On the 30th day of the experiments, the CIA model mice were randomized into 8 groups. G1 and G2 had 12 mice and the other groups had 8 mice. The drugs were subcutaneously injected to the mice. For normal control group and model control group, 0.1 mL/10 g physiological saline was injected once every two days with a total of 8 time injections.

#### 4.5.3. The Assessment of Arthritis

Mouse body weight was measured with an electronic balance once per two days. Paw thickness of left and right hind paws of each mouse was measured with a Vernier caliper once every three days. Joint width of left and right ankles of each mouse was measured with a Vernier caliper once every three days. The arthritis severity was scored by grading of each paw. 0: no erythema or swelling; (1): erythema or swelling of toe joints; (2): erythema and swelling of toes and toe joints; (3): erythema and swelling of ankles; (4): complete erythema and swelling of toes, toe joints and ankles. Scoring was done once every three days, until the end of the experiment.

#### 4.5.4. The Assessment of Pathology

Blood was obtained from orbit and serum was isolated. After the treatment, mice were euthanized. Spleen and thymus were isolated and weighed, then fixed in formalin. The complete paw with ankle was also weighed and fixed into 10% formalin. The fixed paw was decalcified and was embedded in paraffin. The paw sections were then stained with H&E and observed: (1). The presence of hyperplasia of the synovium; (2). The necrosis of the synovium; (3). The presence of pannus formation; (4). The presence of the infiltration of inflammatory cells; (5). The erosion of joint and soft tissue.

### 4.6. Preliminary Pharmacokinetic Study of HM-3-Fc in Monkeys

10 cynomolgus (3 to 5 years of age, 3 to 4 kg of weight, 5 male and 5 female) were randomly divided into 2 groups: Group 1 was comprised of two male and two female cynomolgus, which were treated with HM-3 (0.5 mg/kg); and Group 2 was comprised of three male and three female cynomolgus, which were treated with HM-3-Fc (5.77 mg/kg). Blood samples (approximately 0.8~1 mL) were collected in micro container tubes. Centrifugation at 12,000 rpm for 5 min was operated immediately after heparin anticoagulation and the plasma was collected and stored frozen at −80 °C. For the HM-3 group, blood samples were collected before the injection and 1, 3, 5, 7, 10 h after injection. For the HM-3-Fc group, blood samples were collected before the injection and 0.5, 1, 2, 3, 4, 8, 12, 16, 20, 24, 36, 48, 60, 72, 84, 96, 120, 144, 168, 192, 216, 240 h after injection. The plasma concentrations of HM-3 and HM-3-Fc were determined using competitive ELISA (Nanjing Anji Biotechnology Co., Ltd., Nanjing, China). The minimum concentration which can be detected in this method was 100 ng/mL and the precisions within and between the groups were 4.41 to 6.86% and 6.78 to 9.36%, respectively.

### 4.7. Statistically Analysis

The data were analyzed by SPSS 19.0 (SPSS Inc., Chicago, IL, USA). The results were indicated by mean ± SD. The one-way analysis of variance (ANOVA) was used for group comparison. For all statistical analyses, a *p* value of less than 0.05 was considered statistically significant and that of less than 0.01 was considered statistically very significant.

## Figures and Tables

**Table 1:** Strategy of collagen-induced arthritis (CIA) model mice treatments.

Group Name	Immunization or Not	Treatment Drugs	Way of Administration
G1 (*n* = 12)	no		
G2 (*n* = 12)	yes		
G3 (*n* = 8)	yes	Adalimumab	8 mg/kg, subcutaneous (s.c.), once every two weeks
G4 (*n* = 8)	yes	HM-3	1.6 mg/kg, s.c., twice a day
G5 (*n* = 8)	yes	HM-3-Fc	50 mg/kg, s.c., once every five days
G6 (*n* = 8)	yes	HM-3-Fc	25 mg/kg, s.c., once every five days
G7 (*n* = 8)	yes	HM-3-Fc	12.5 mg/kg, s.c., once every five days
G8 (*n* = 8)	yes	HM-3-Fc	25 mg/kg, s.c., once every seven days

**Table 2:** The changes of mice paw thickness, tarsus width and paw perimeter on the last day of the experiment. (X ± SD, *n* = 12 or 8, mm).

Group	G1	G2	G3	G4	G5	G6	G7	G8
Paw thickness	2.46 ± 0.13	3.85 ± 0.30 ^##^	3.04 ± 0.11	3.06 ± 0.23	2.92 ± 0.11	2.94 ± 0.07	3.15 ± 0.07	2.99 ± 0.17
Tarsus width	3.48 ± 0.12	4.83 ± 0.20 ^##^	4.23 ± 0.08	4.21 ± 0.15	3.96 ± 0.09 *	3.98 ± 0.08 *	4.26 ± 0.13	4.00 ± 0.16
Paw perimeter	9.32 ± 0.28	13.62 ± 0.64 ^##^	11.42 ± 0.20 **	11.41 ± 0.53 **	10.80 ± 0.26 **	10.87 ± 0.14 **	11.64 ± 0.19 **	10.97 ± 0.32 **

^##^*p* < 0.01 vs G1 (normal control group). * *p* < 0.05, ** *p* < 0.01 vs. G2 (model control group).

**Table 3:** Pharmacokinetic parameters of HM-3 and HM-3-Fc in cynomolgus ^a^.

	HM-3 0.5 mg/kg	HM-3-Fc 5.77 mg/kg
Half-life (h)	0.022 ± 0.013	15.24 ± 2.32 * ^b^
C_max_ (ng/mL)	1819.52 ± 435.91	56,112.3 ± 12,477.4 *
AUC_0-240_ (h·ng/mL)	64.29 ± 27.5	722.89 ± 187.86 *

^a^ Data were shown as mean ± SEM. ^b^ * *p* < 0.05, vs. HM-3 0.5 mg/kg.

## Supplementary Materials

Supplementary materials can be found at http://www.mdpi.com/1422-0067/19/9/2683/s1.
