# Supplementary material for: The Protective Effect of a Long-Acting and Multi-Target HM-3-Fc Fusion Protein in Rheumatoid Arthritis
Source: Int J Mol Sci. 2018 Sep 10;19(9):2683. doi: 10.3390/ijms19092683 (PMC6163367; doi:10.3390/ijms19092683)
Supplement: Supplementary file 1 [file ijms-19-02683-s001.pdf]

Supplementary Materials

1. Purification of HM-3-Fc after cell culturing

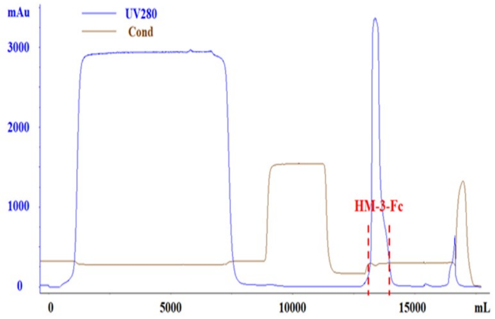

Supplementary Figure S1. Elution profile of culture supernatant through Protein A affinity chromatography. 6.23 L culture supernatant was filtered through a 0.45 um filter and was loaded onto a Protein A affinity column (XK50/20, 294 mL, 15 cm) with a flow rate of 20 ml/min at 4℃. After brief washing, HM-3-Fc was eluted by 50 mM Citric/Citrate, 0.15 M NaCl, pH 3.0. A single peak with high protein concentration was obtained after elution with a shoulder peak.

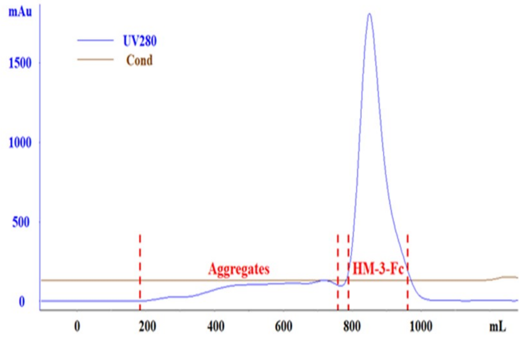

Supplementary Figure S2. Elution profile of the elution fraction from Protein A affinity column through size exclusion chromatography. 65 ml Protein A column elution sample was loaded onto Sephadex 200 size exclusion chromatography (XK50/100, Volume 1531 mL, Height 78 cm) with a flow rate of 8 mL/min. A single peak with high protein concentration was obtained. Quality control experiments of the component in the main peak were performed.

2. Effect of HM-3-Fc on splenocyte viability

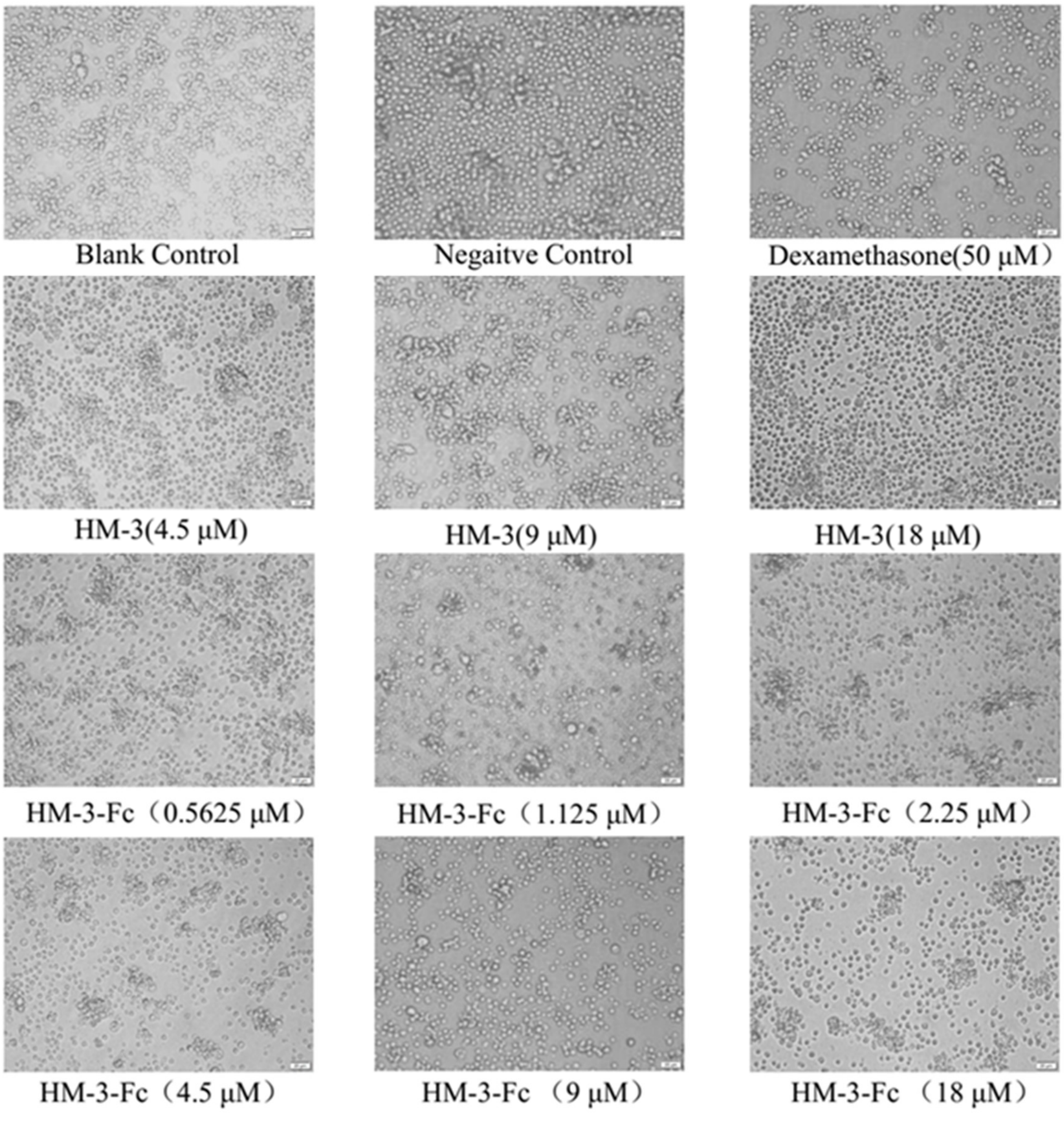

Supplementary Figure S3. Photographs of the splenocytes before or after HM-3-Fc treatment. Freshly prepared splenocytes were diluted to a final concentration of 2×10^6 cells/mL and were cultured in flat-bottom 96-well plates (100uL per well) in presence of 5 μg/mL/well ConA. A well with cells without ConA stimulation was used as a blank well. At the same time, 90 μL of the following drugs were added: the control group containing 100 μL media, Dexamethasone (50 μmol/L), HM-3-Fc (0.5625, 1.125, 2.25, 4.5, 9, 18 μmol/L).

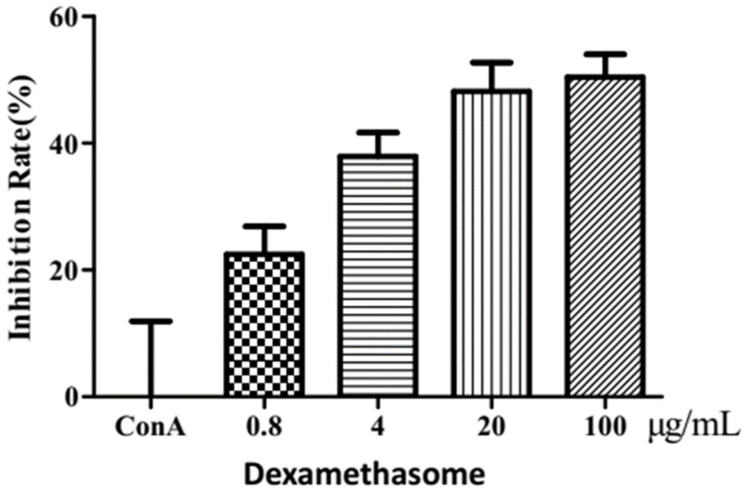

Supplementary Figure S4. Effect of Dexamethasome on viability of splenic lymphocytes. Fresh isolated splenic lymphocytes were stimulated with 0.5 μg/mL ConA in absence or presence of 0.8, 4, 20 or 100 μg/mL Dexamethasome. Inhibitory effect of Dexamethasome on splenic lymphocyte viability was evaluated in comparison with ConA stimulated sample.

3. Cytotoxic effect of HM-3-Fc on macrophage U937 cells

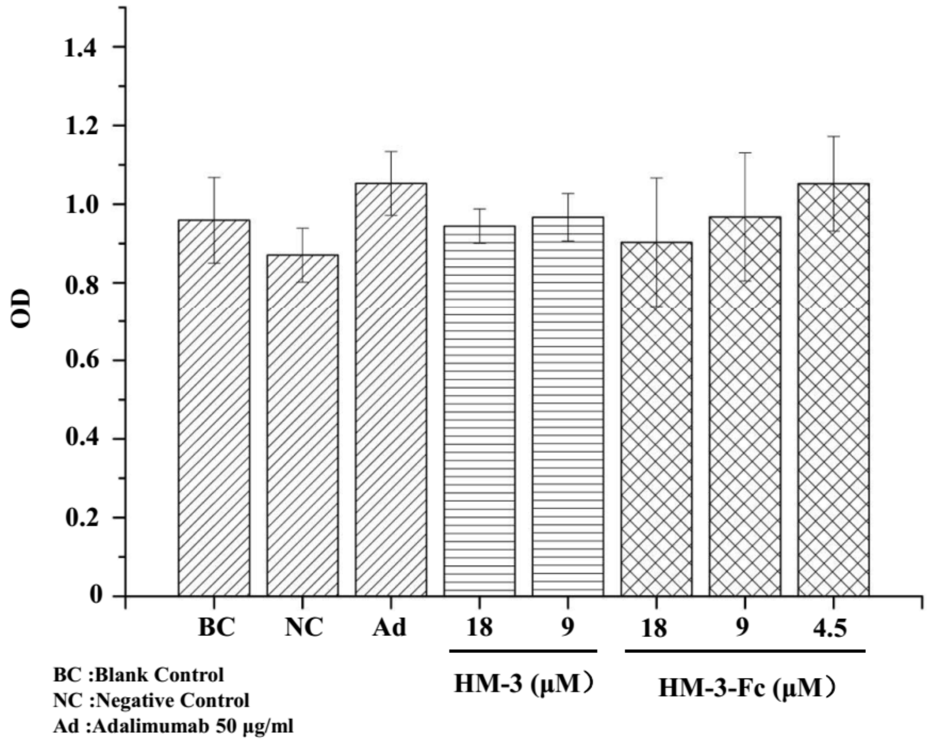

Supplementary Figure S5. MTT method to evaluate the cytotoxic effect of HM-3-Fc on macrophage U937 cells. After drug treatment for 48 hours, cell supernatant was collected for TNF-α detection and the same volume of fresh culture medium was added. Then, 20 μL MTT (5 mg/mL) was added in each well and the plate was incubated in the incubator for another 4 hours. The media solution in the plate was discarded, and 100 μL DMSO was added to each well and mixed gently. The conversion of MTT to formazan in metabolically viable cells was spectrophotometrically measured at 570 nm with 630 nm as a reference wavelength.

4. The effects of HM-3-Fc on mice paw

4.1. Paw thickness

The thickness of left and right hind paws of each mouse was measured with vernier caliper once per three days. From Supplementary Figure 6A, paw thickness of model control group (3.32 ± 0.35 mm) showed great increase compared with that of G1 group (normal control group) (2.51 ± 0.17 mm), with significant difference (**$p<0.01$). Drug treatments resulted in a reduction of paw thickness. Adalimumab (G3 group) was administered with a single subcutaneous injection at 2 week's interval and the paw thickness in G3 showed significant reduction from Day 39 (*$p<0.05$). Mice in G4 group were administered with HM-3 twice a day via subcutaneous injection for 15 days at a dose of 1.6 mg/kg and showed significant reduction of paw thickness from Day 51 (*$p<0.05$). Mice in G5-G7 were administered with HM-3-Fc once every 5 days via subcutaneous injections for 15 days at a dose of 50 mg/kg (G5), 25 mg/kg (G6) or 12.5 mg/kg (G7). Mice in G5 showed significant reduction of paw thickness from Day 42 (*$p<0.05$). Mice in G6 showed significant reduction of paw thickness from Day 45 (*$p<0.05$). Mice G7 showed significant reduction of paw thickness from Day 48 (*$p<0.05$). And mice in G8 were administered with HM-3-Fc once every 7 days via subcutaneous injection for 15 days at a dose 25 mg/kg and showed significant reduction of paw thickness from Day 48 (*$p<0.05$) (Supplementary Figure 6A).

4.2. Paw thickness

The tarsus width of left and right hind ankles of each mouse was measured with vernier caliper once every three days. The tarsus width of model control group mice (4.34±0.40 mm) was obviously wider than that of normal control group mice (3.43±0.14 mm) with significant difference (** $p<0.01$). Drug treatments resulted in reduction of tarsus width. Mice in G3 showed significant decrease of tarsus width from Day 36 (* $p<0.05$). Mice in G4 showed significant decrease of tarsus width from Day 48 (* $p<0.05$). Mice in G5 showed significant decrease of tarsus width from Day 42 (* $p<0.05$). Mice in G6 showed significant decrease of tarsus width from Day 45 (* $p<0.05$). Mice in G7 showed significant decrease of tarsus width from Day 45 (* $p<0.05$). And mice in G8 showed significant decrease of tarsus width from Day 45 (* $p<0.05$) (Supplementary Figure 6B).

4.3. Paw perimeter

The paw perimeter of mice in model control group (12.03±0.96 mm) was obviously longer than that of normal control group mice (9.34±0.33 mm) with significant difference (** $p<0.01$). Drug treatments resulted in reduction of paw perimeter. Mice in G3 showed significant decrease of paw perimeter from Day 39 (* $p<0.05$). Mice in G4 showed significant decrease of paw perimeter from Day 51 (* $p<0.05$). Mice in G5 showed significant decrease of paw perimeter from Day 42 (* $p<0.05$). Mice in G6 showed significant decrease of paw perimeter from Day 45 (* $p<0.05$). Mice in G7 showed significant decrease of paw perimeter from Day 48 (* $p<0.05$). And mice in G8 showed significant decrease of paw perimeter from Day 48 (* $p<0.05$) (Supplementary Figure 6C).

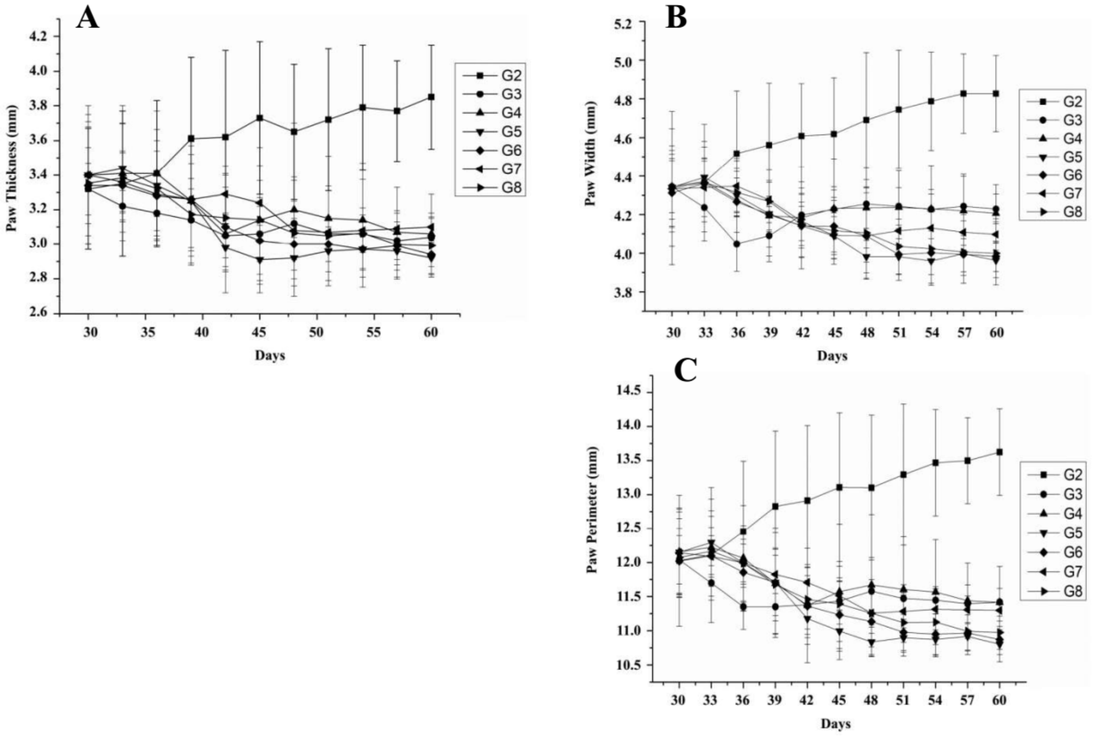

Supplementary Figure S6. HM-3-Fc treatments decreased paw thickness, tarsus width, and_paw perimeter of CIA mice. Mice were immunized with an emulsion of bull collagen type II and Complete Freund's adjuvant (CFA) on day 0 and subsequently with an injection of incomplete Freund's adjuvant (IFA) on day 21. On the 30th day of the experiments, mice were randomized in different groups for treatments with different drugs. Paw thickness (A), tarsus width (B) and paw perimeter (C) of mice in each group were measured every three days until the 60th day. For G2, n=12; for G3-G8, n=8.

5. Arthritis grading of CIA mice after HM-3-Fc treatments

Dynamic change of arthritis grading of CIA mice in each group was shown in Figure 7A. Mice in G3 showed significant decrease in arthritis grading from Day 39 (**p<0.05). Mice in G4 showed significant decrease of arthritis grading from Day 48 (**p<0.05). Mice in G5 showed significant decrease of arthritis grading from Day 39 (**p<0.05). Mice in G6 showed significant decrease of arthritis grading from Day 45 (**p<0.05). Mice in G7 showed significant decrease of arthritis grading from Day 51 (**p<0.05). And mice in G8 showed significant decrease of arthritis grading from Day 45 (**p<0.05).

6. Effects of HM-3-Fc on the weights of spleen, thymus, paw and body weight of CIA mice

6.1. Spleen

The spleen weight of G2 model mice (114.6±14.5 mg) had very significant difference compared with G1 normal mice (74.5±14.4 mg, **p <0.01). The spleen weight of mice in G5 had very significant difference compared with G2 (**p <0.01). The spleen weight of mice in G3, G4, G6, G7, G8 had significant difference compared with that of G2 (**p <0.05).

6.2. Thymus

The thymus weight of G2 model mice (39.9±8.9 mg) had significant difference compared with G1 normal mice (25.9±6.2 mg, **p <0.05). Mice in all the other groups had no significant difference compared with G2 model mice.

6.3. Paw weight

On the 60th day of the experiment, mice in each group were athesthized and killed. Right and left paws of each mice in each group were weighted and compared. The paw weight of mice in G2 model group (219.3 ± 20.6 mg) had very significant difference compared with mice in G1 normal control group (159.0 ± 6.6 mg, **p <0.01). Paw weight of mice in G5 (176.2 ± 8.9 mg), G6 (176.4 ± 9.7 mg) and G8 (177.1 ± 7.8 mg) had very significant difference (**p<0.01) compared with that of G2 (219.3 ± 20.6 mg). The paw weights of G3 (181.9 ± 16.2 mg), G4 (185.6 ± 16.1 mg) and G7 (188.0 ± 15.8 mg) had significant difference (**p <0.05) compared with that of G2 (219.3±20.6 mg).

6.4. Body weight

The body weight of mice in G2 model group (19.7 ± 0.9 g) had very significant difference compared with that of mice in G1 normal control group (22.0 ± 1.1 g, **p <0.01). After drug treatments, the weights of mice in each group gradually increased. On Day 60 of the experiment, the body weight of mice in G3, G4, G5, G6, G7, G8 groups were 21.0 ± 1.5, 20.9 ± 1.5, 20.8 ± 1.8, 21.1 ± 1.6, 21.5 ± 1.5g, 20.8 ± 1.7g, respectively. Body weights of mice in drug treatments groups gradually increased compared with that of model control group mice (19.5 ± 1.6 g, *p<0.05).

All the results above led to the conclusion that, with different doses and frequencies of HM-3-Fc treatments, the mice showed significant difference in spleen, paw and body weight, but no difference in thymus weight was found compared with model control group mice (Supplementary Table 1).

Supplementary Table S1. The thymus, spleen, paw and body weight of the mice on the last day of the experiment（X ± SD, n=12 or 8 mg）

**Group**	**G1**	**G2**	**G3**	**G4**	**G5**	**G6**	**G7**	**G8**
Thymus(mg)	25.9±6.2	39.9±8.9##	31.2±9.5*	31.2±6.7*	33.4±6.6	37.1±7.0	38.1±11.3	38.3±9.1
Spleen(mg)	74.5±14.4	114.6±14.5##	94.6±6.7**	94.2±13.3**	90.6±7.7**	95.1±9.5**	97.5±5.1**	95.8±11.4*
Paw(mg)	159.0±6.6	219.3±20.6##	181.9±16.2**	185.6±16.1**	176.2±8.9**	176.4±9.7**	188.0±15.8**	177.1±7.8**
Body(g)	22.1±1.3	19.5±1.6##	21.0±1.5*	20.9±1.5	20.8±1.8	21.1±1.6*	21.5±1.5**	20.8±1.4

#p<0.05，##p<0.01 vs G1（normal control group）. *p<0.05，**p<0.01 vs G2（model control group）.
